# Relevant patient characteristics for guiding tailored integrated diabetes primary care: a systematic review

**DOI:** 10.1017/S146342361800004X

**Published:** 2018-02-06

**Authors:** Dorijn F.L. Hertroijs, Arianne M.J. Elissen, Martijn C.G.J. Brouwers, Nicolaas C. Schaper, Dirk Ruwaard

**Affiliations:** 1 Department of Health Services Research, CAPHRI Care and Public Health Research Institute, Faculty of Health, Medicine and Life Sciences, Maastricht University, Maastricht, The Netherlands; 2 Department of Internal Medicine, Division of Endocrinology, CARIM School for Cardiovascular Diseases, Maastricht University Medical Centre, Maastricht, The Netherlands; 3 Department of Internal Medicine, Division of Endocrinology, CAPHRI School for Public Health and Primary Care, Faculty of Health, Medicine and Life Sciences, Maastricht University Medical Center, Maastricht, The Netherlands

**Keywords:** integrated health-care systems, patient-centered care, primary care, review, type 2 diabetes mellitus

## Abstract

**Aim:**

To identify which patient-related effect modifiers influence the outcomes of integrated care programs for type 2 diabetes in primary care.

**Background:**

Integrated care is a widespread management strategy for the treatment of type 2 diabetes. However, most integrated care programs are not tailored to patients’ needs, preferences and abilities. There is increasing consensus that such a patient-centered approach could improve the management of type 2 diabetes. Thus far, it remains unclear which patient-related effect modifiers should guide such an approach.

**Methods:**

PubMed, CINAHL and EMBASE were searched for empirical studies published after 1998. A systematic literature review was conducted according to the PRISMA guidelines.

**Findings:**

In total, 23 out of 1015 studies were included. A total of 21 studies measured the effects of integrated diabetes care programs on hemoglobin A1c (HbA1c) and three on low-density lipoprotein cholesterol, systolic blood pressure and health-care utilization. In total, 49 patient characteristics were assessed as potential effect modifiers with HbA1c as an outcome, of which 46 were person or health-related and only three were context-related. Younger age, insulin therapy and longer disease duration were associated with higher HbA1c levels in cross-sectional and longitudinal studies. Higher baseline HbA1c was associated with higher HbA1c at follow-up in longitudinal studies. Information on context- and person-related characteristics was limited, but is necessary to help identify the care needs of individual patients and implement an effective integrated type 2 diabetes tailored care program.

## Introduction

Diabetes is one of the most prevalent chronic conditions worldwide and a public health priority in many countries (Tamayo *et al*., 2014; International Diabetes Federation, 2015). In Europe, an estimated 9.8 million people suffer from diabetes; type 2 diabetes is responsible for 90% of cases. People with type 2 diabetes are at high risk for developing complications, such as cardiovascular disease and kidney failure, which in turn lead to increased health-care costs (Tamayo *et al*., 2014; International Diabetes Federation, 2015). To prevent diabetes-related co-morbidities and complications, and lower medical care expenditure for patients with type 2 diabetes, it is important to implement effective and efficient management strategies. An example of such a strategy is the implementation of integrated care. It aims to improve patient care and experience through improved coordination (Shaw *et al*., 2011).

The implementation of integrated care programs is widespread in North America, Europe, and other parts of the world (Kodner, 2009; Shaw *et al*., 2011). However, most integrated care programs are not tailored to patients’ needs and preferences, but rather highly standardized according to evidence-based guidelines for specific diseases, such as diabetes. Findings from recent studies suggest that not all patients benefit equally from such a standardized approach (Rothe *et al*., 2008; Pimouguet *et al*., 2011; Elissen *et al*., 2012). These studies report that patients with poorly controlled diabetes benefit mostly from intensive, provider-driven disease management, whereas patients with adequate glucose levels might maintain these levels independent of the type of care they receive.

In 2012, the European Association for the Study of Diabetes and the American Diabetes Association recommended a more patient-centered approach for the management of type 2 diabetes (Inzucchi *et al*., 2012). In a patient-centered approach, care is tailored according to individual patient needs and preferences (Commitee on Quality of Health Care in America; Institute of Medicine, 2001; Inzucchi *et al*., 2012; American geriatrics society expert panel on person-centered care, 2016; Coulourides Kogan *et al*., 2016). It draws on the concept of ‘mass customization’, where goods and services are delivered with enough variety and customization that nearly everyone finds exactly what they want (Tseng and Hu, 2014). Dividing the population based on health-care needs creates groups that are more homogenous than the population as a whole. Hence, care offered to these groups will be more tailored to the patients’ needs, while acknowledging that a certain amount of heterogeneity within the subgroups will remain.

There is increasing consensus that a patient-centered approach could improve the management of type 2 diabetes (Inzucchi *et al*., 2012). However, to date, it is unclear what the best method is for establishing patient-centered care (Epstein and Street, 2011). Since intensive, provider-driven disease management is not beneficial to every type 2 diabetes patient, several studies have pointed toward patient characteristics – for example, number of co-morbidities, disease duration or attitude – as possible effect modifiers of treatment (Hasnain-Wynia and Baker, 2006; Inzucchi *et al*., 2012; Riddle and Karl, 2012; Scheen, 2016). These effect modifiers could be used to identify patients with different care needs and preferences, and subsequently serve as input to tailor treatment (Goldberger and Buxton, 2013; Constand *et al*., 2014) . However, it is unclear which effect modifiers should guide a more patient-centered approach. Therefore, the aim of this systematic review was to identify which patient effect modifiers influence the outcomes of integrated care programs for type 2 diabetes in primary care. These effect modifiers can help to segment the chronically ill population into subgroups with similar health-care needs for whom, based on insight into their needs and preferences, a range of matching care and support options can be developed.

This review is the first part of the research project entitled ‘PROFiling patients’ healthcare needs to support Integrated, person-centered models for Long-term disease management (PROFIle)’ (Elissen *et al*., 2016). The aim of this four-year Dutch project is explicitly not to develop another disease-specific approach, but we use type 2 diabetes as starting point to develop, validate and test so-called ‘patient profiles’ as an instrument to support more patient-centered chronic care management in practice.

## Methods

### Data sources and searches

A systematic literature search according to PRISMA guidelines (Moher *et al*., 2009) was performed on PubMed, CINAHL and EMBASE databases in January 2015. Included were English- or Dutch-language randomized controlled trials (RCT), prospective and retrospective cohort- and cross-sectional studies which: (1) focused on integrated care (defined below); (2) included adult patients (⩾18 years) with type 2 diabetes; (3) were set in primary care; (4) measured effects on 1 or more measures of diabetes management [hemoglobin A1c (HbA1c), low-density lipoprotein cholesterol (LDL-c) and systolic blood pressure (SBP)], and/or health-care utilization as outcome variables; and (5) included sub-analyses with patient characteristics as independent variables. In line with previous research, integrated care was defined as interventions combining two or more components of the well-known Chronic Care Model (CCM) (Busetto *et al*., 2016). The CCM stresses the need for a more proactive health-care system by focusing on four components: self-management support (eg, patient education), decision support (eg, evidence-based guidelines), delivery system design (eg, care process) and clinical information systems (eg, electronic registries) (McCulloch *et al*., 1998; Coulter *et al*., 2015). Since the CCM was developed in 1998, only studies published in or after 1998 were included (Austin *et al*., 2000). The search strategy included targeted terms related to diabetes, integrated care, CCM components, care outcomes and subgroup analyses based on patient characteristics. The complete search terms and search string can be found in Table 1. The snowball method was used to search for other relevant studies.

**Table 1:** Search terms and search string

#	Category	Search terms
1	Diabetes	Diabetes OR diabetes mellitus OR diabetic patient OR type 2 diabetes OR type 2 diabetes mellitus OR T2DM OR NIDDM
2	Integrated care	Integrated care OR disease management OR disease state management OR comprehensive healthcare OR comprehensive health care OR shared care OR coordinated care OR case management OR chronic care model OR primary care OR primary health care OR outpatient clinic OR outpatient services OR primary health care OR primary healthcare OR primary health clinics OR general practice OR family practice OR community care
3	CCM – self-management support	Self-management OR self-management support OR self-care OR patient-centeredness OR patient-centered care OR behavioral support OR motivational support OR self-management education OR patient education
4	CCM – delivery system design	Delivery system design OR care pathway OR critical pathway OR individualized care OR clinical case management OR medicines management OR medication management OR comorbidities management OR health literacy OR cultural sensitivity OR practice nurse OR care team OR health care team Or healthcare team OR patient care team OR personalized care OR personalized management OR individualized management OR multidisciplinary care team OR tailored care OR tailored support OR multidisciplinary care
5	CCM – decision support	Decision support, clinical reminders, clinician reminders, patient reminders, provider education, reminder systems, individualized care plans, individual care plans
6	CCM – clinical information system	Clinical information system, clinical information systems, clinical registry, health information system, health information systems, health information technology, electronic registry, clinical reminders, clinician reminders, patients reminders, provider feedback, performance monitoring, ICT device, patient portal, patient registry, diabetes registry, telemonitoring, telehealth, teleassistance, telehomecare, videoconferencing, mobile phone
7	Outcome measures	Glycemic control, glycaemic control, diabetic control, diabetes control, diabetes status, Charlson Comborbidity Index, resource use, health care use, health care utility, service use, resource utility, service utility
8	Subgroup analysis	Factor, predictor, predictive factor, determinant, patient characteristic, patient characteristics, patient feature, patient features, patient dynamics, subgroup, subgroups, segment, strata, classes
9	Complete search string	#1 AND (#2 OR (#3 AND #4) OR (#3 AND #5) OR (#3 AND #6) OR (#4 AND #5) OR (#4 AND #6) OR (#5 AND #6)) AND #7 AND #8

CCM=Chronic Care Model.

### Study selection

Potentially relevant studies were retrieved from the electronic databases based on the inclusion criteria in three screening rounds. First, titles and abstracts were screened. The first 50 titles and abstracts were screened independently by two reviewers (D.H. and A.E.). More than 90% agreement was reached. Therefore, the remainder of the titles and abstracts were screened by 1 reviewer (D.H.). Second, the first 20 full texts were screened independently by two reviewers (D.H. and A.E.). Again, more than 90% agreement was reached and therefore, each reviewer independently screened half of the full texts. Third, the reference lists of the included studies were screened to obtain additional studies. Steps 1 and 2 of the study selection process were then repeated.

### Data extraction and quality assessment

Descriptive data on studies were extracted by 1 reviewer (D.H.) between August and October 2015. Studies were coded for author names, year of publication, country, study design, length of follow-up, population size, age, percentage of males and CCM components. In case of uncertainties, a group discussion was held with two other authors (A.E. and M.B.).

The Effective Public Health Practice Project Quality Assessment Tool (EPHPP) was used to assess the quality of the included studies (Armijo-Olivo *et al*., 2012). This tool was chosen because it allows the assessment of different study designs. The studies were rated based on six domains: (1) selection bias; (2) study design; (3) confounders; (4) blinding; (5) data collection; and (6) withdrawals and dropouts. Each domain was rated as ‘strong,’ ‘moderate’ or ‘weak’. A global rating was given based on the number of weak components.

Two reviewers (D.H. and M.B.) independently performed the quality assessment for each study. Disagreements were resolved via discussion conform EPHPP guidelines.

### Data synthesis and analysis

The included studies were categorized according to: (1) the reported outcome(s) of interest (HbA1c, LDL-c, SBP and/or health-care utilization); and (2) the type of patient characteristic(s) investigated in subgroup analyses. Characteristics were classified as person-related (predisposing), context-related (enabling) or health-related (illness level) characteristics according to Andersen and Newman’s (1973) Behavioral Model of Health Service Use. The model provides a theoretical framework for viewing health services utilization, taking into account both societal and individual characteristics. The model was chosen, because the individual characteristics can inform tailored care by, for example, helping determine the best intensity of care for the individual patient. Relationships between outcomes and characteristics were depicted as ‘+’ for significant positive relationships, as ‘−‘for significant negative relationships and as ‘o’ for non-significant relationships.

## Results

### Search results

**Figure 1:**
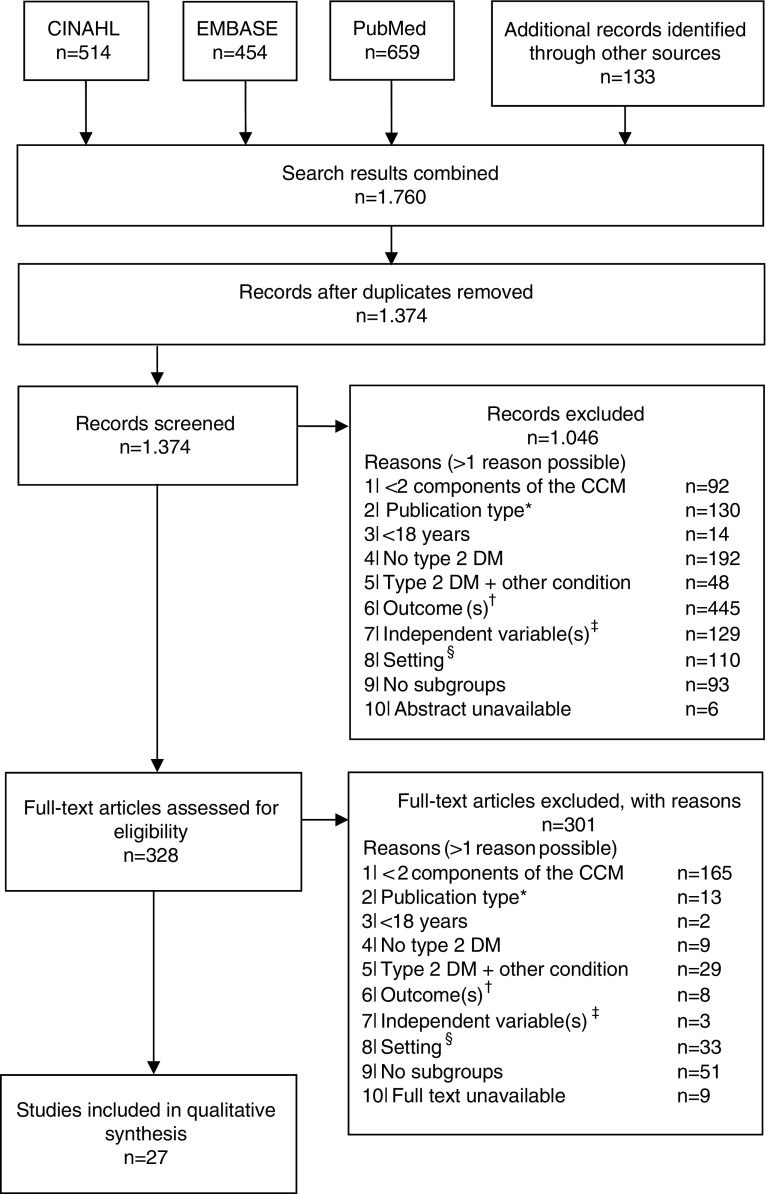
Flow diagram of the study selection. *Qualitative, or mixed-method studies; ^†^any outcome other than hemoglobin A1c, low-density lipoprotein cholesterol, blood pressure or health-care utilization; ^‡^independent variable is not a person-, context- or health-related patient characteristic (eg, health-care provider characteristics); ^§^setting is not a primary care setting (eg, hospital). CCM=Chronic Care Model; DM=diabetes mellitus.

In total, 1374 studies were identified through electronic databases and by checking the references of the included studies. Figure 1 shows the flow diagram of the study selection. Most studies were excluded because none relevant outcomes were reported (*n*=453), and/or type of care was not integrated (*n*=257). After the title, abstract and full text screening, 27 studies were included (Groeneveld *et al*., 2001; Ostgren *et al*., 2002; El-Kebbi *et al*., 2003; Rothman *et al*., 2003; Rothman *et al*., 2004; Uitewaal *et al*., 2004; Benoit *et al*., 2005; Sperl-Hillen and O’Connor, 2005; Uitewaal *et al*., 2005; De Alba Garcia *et al*., 2006; Nielsen *et al*., 2006; Taweepolcharoen *et al*., 2006; Trief *et al*., 2006; Wahba and Chang, 2007; Mold *et al*., 2008; Al Omari *et al*., 2009; De Fine Olivarius *et al*., 2009; Robinson *et al*., 2009; Kellow *et al*., 2011; Cardenas-Valladolid *et al*., 2012; Elissen *et al*., 2012; Liu *et al*., 2013; Quah *et al*., 2013; LeBlanc *et al*., 2015; Luijks *et al*., 2015; Moreira *et al*., 2015; Quinn *et al*., 2016).

### Quality assessment

The methodological quality of the included studies can be found in Supplementary Table 1. The domains with the most ‘weak’ ratings were confounders (*n*=10), blinding (*n*=9) and selection bias (*n*=9). Almost all studies (*n*=25) scored high on the domain data collection. The overall study quality was strong for four studies, moderate for 11 studies and low for 12 studies. Most studies with low quality had a cross-sectional study design and did not report on or adjust for possible confounders.

### Study and sample characteristics

Of the included studies, nine (33.3%) were retrospective cohort studies, seven (25.9%) cross-sectional studies, seven (25.9%) (randomized) controlled studies and four (14.8%) prospective cohort studies. Table 2 shows that the median follow-up duration for retrospective cohort, prospective cohort and randomized controlled studies (*n*=20) was 15 months (range 6–112). The median sample size consisted of 376 individuals (range 80–105 056) with an average age of 60.0 years (range 50.5–70.9); the percentage of male subjects ranged from 31.3 to 68.0.

**Table 2:** Study and sample characteristics

Study characteristics				Sample characteristics			CCM			
							Description of components			
Study	Country	Study design	Follow-up (months)	*n*	Age (SD or range)	Sex (% male)	Self-management support	Delivery system design	Clinical information systems	Decision support
Al Omari *et al*. (2009)	JOR	CS	N/A	337	54.1 (11.3)	52.1	Regular group counseling with the presence of family physicians, nurses, pharmacists and dieticians Leaflets related to diabetes	Care team (doctor and diabetic nurse) Regular follow-up: patient has to see the physician to take the prescription on a monthly basis		
Benoit *et al*. (2005)	USA	RC	24	573	55.4 (10.1)	31.3		The nurse educator is the case manager Nurse educator identifies individual service and access needs of patients Nurse communicates with the primary care physician regarding clinical issues	Nurse educator follows up on missed patient appointments Diabetes electronic medical system software	
Cardenas-Valladolid *et al*. (2012)	ES	PC	24	23 488	69.7 (14.5)	48.4	Interventions focused on drug therapy compliance, change in lifestyle, health education and self-management		Computerized clinical record	
De Fine Olivarius *et al*. (2009)	DK	PC	66	581	64.7 (55.7–73.2)	51.9	Individualized goal setting	Follow-up every 3 months annual screening for diabetic complications	Annual descriptive feedback reports on individual patients	Clinical guidelines supported by annual half day seminar
Elissen *et al*. (2012)	NL	RC	20–24	105 056	65.7 (11.9)	Unknown	National Diabetes Care Standard includes general modules on information, education and self-management support, smoking, cessation, physical activity, nutrition and diet	Care team (GP, practice nurse)	Shared diabetes patient registry	Defined frequency of GP visits, regular foot and eye examinations, laboratory testing
El-Kebbi *et al*. (2003)	USA	RC	5–12	2539	55.0 (12.0)	44.0	Education program emphasizing lifestyle modifications and self-management skills offered to all patients at their initial visit and projects 6 to 8 return visits within the first year	Patients cared for by a team of nurse providers, physicians, dietitians, podiatrists and a social worker		If glycemic goals are not met after the first one to two months, pharmacologic therapy is started or advanced according to a stepped-care protocol for intensification of therapy
De Alba Garcia *et al*. (2006)	Mex	CS	N/A	796	60.5 (10.8)	38.6	Diabetes and nutrition education Diabetes and exercise support groups	Care team (physicians, nutritionist and psychologist)		
Groeneveld *et al*. (2001)	NL	RCT	12	I: 91 C: 155	I: 62.7 (11) C: 62.3 (10)	I: 34.1 C: 46.4	Counseling by a diabetes educator (nurse) and dietician at the ‘Diabetes Service’, a monitoring and advisory service	Care team consisting of diabetes educator (nurse), dietician and GP Patients were called up and reviewed every three months. If insulin was started contacts were more frequent		GP responsible for implementation of therapeutic advice of the Diabetes Service
Kellow, Savige and Khalil (2011)	AUS	RC	60	272	62.1 (11.6)	49.0	Diabetes education at the health service diabetes education department	Care team (GP, diabetes educator). Diabetes educator referred patients for additional optometry, podiatry and dietetic appointments as required		
LeBlanc *et al*. (2015)	USA	RC	12	14 430	63 (55.0–76.0)	52.5			Electronic medical record system	Evidence-based treatment guidelines
Liu *et al*. (2013)	CH	CS	N/A	960	68.3 (10.4)	39.6		Health management Follow-up every three months		Community diabetes prevention and treatment guidelines provide glycemic control targets
Luijks *et al*. (2015)	NL	PC	60	610	63 (12.5)	48.2		Routine three-monthly check-up visits	Electronic medical record system	
Mold *et al*. (2008)	UK	RC	11	646	<50: 16.4% 50–59: 18.3% 60–69: 31.1% ⩾70: 34.2%	54.3	Dietary advice is offered at each consultation	Care team (GP, practice nurse) Patients initially see the GP and are then referred to the practice nurse	Electronic medical record system	
Moreira *et al*. (2015)	Brazil	RCT	12	I: 40 C: 40	I: 50.0 (6.5) C: 50.3 (7.3)	I: 40 C: 30	Educational activities focused on providing orientation about physical activities, healthy diet, monitoring capillary glycemia, and acute and chronic complications	Quarterly nursing consultations, bimonthly educational group activities. When necessary referral for a consultation with a primary health-care physician, nurse, nephrologist, pharmacist and nutritionist. Home visits and phone contacts on a monthly basis with the case manager		
Nielsen *et al*. (2006)	DK	RCT	72	I: 459 C: 415	Median I: 63.0 (53.8–71.4) C: 63.7 (65.6–71.6)	I: 48.8 C: 52.3	Individualized goal setting	Follow-up every three months Annual screening for diabetic complications	Annual descriptive feedback reports on individual patients	Clinical guidelines supported by annual half day seminar
Óstgren *et al*. (2002)	SWE	CS	N/A	376	HbA1c<6.5: 69.6 (10.4) HbA1c⩾6.5: 70.9 (9.8)	50.5	Structured education program	Specially trained nurses, supervised by the physician. Team also included a dietician and a podiatrist		Structured treatment program, including annual check-up at hypertension and diabetes outpatient clinic including examinations concerning vision, peripheral sensibility of vibration and peripheral pulsation and laboratory tests
Quah *et al*. (2013)	SG	CS	N/A	688	62.2 (11.1)	44.0		Routine three-monthly visit to polyclinics	Diabetes database	
Quinn *et al*. (2016)	USA	RCT	12	118	Age <55 years: I: 47.3 (6.8) C: 47.5 (7.5) Age⩾55 years: I: 59.0 (2.9) C: 59.5 (2.8)	Age <55 years: I: 37.3 C: 62.1 Age⩾55 years: I: 68.0 C: 37.0	Mobile diabetes management software application, which allowed patient to enter diabetes self-care data on a phone and receive automated, real-time messages that were educational, behavioral, motivational and specific to the entered data Electronic diabetes self-care action plan	Patients could communicate with ‘virtual’ case managers on the phone or electronically	Quarterly online reports that summarized patients’ glycemic and metabolic control, etc.	Clinical guidelines
Robinson *et al*. (2009)	USA	PC	18	315	64.4 (15.8)	41.9	Self-monitoring of blood glucose, foot care, diet and exercise modification, diabetes education resources, and participation in planned visits, were addressed through individual and small group appointments with members of the care team and through population-based quality improvement projects All patients in the intervention group were targeted for individual coaching in self-management activities by the NP or pharmacy student	Care team consisting of medicine resident, nurse practitioner students and pharmacy students All participated in chronic illness curriculum Patients seen in individual 30-minute appointments by one or more of the team members Follow-up appointments were scheduled	An electronic clinical information system supplied clinical data	Care team participates in 60-minute didactic presentation, 30-minute clinical discussion session focusing on patient management and quality improvement Weekly presentation topic covered various aspects of diabetes care
Rothman *et al*. (2003)	USA	RC	6	138	57.0 (23–87)	41.0	Diabetes education: 1-h educational session	Three pharmacists participated in the program. Referrals for ophthalmology, nutrition and podiatry also were suggested to the patient and provided when appropriate All recommendations discussed with primary care provider	Computer database Patients were contacted approximately every 2 weeks through phone calls, letters or pharmacy visits	Algorithms for titrating insulin and metformin
Rothman *et al*. (2004)	USA	RCT	12	I: 98 C: 95	I low literacy: 57 (10.5) I high literacy: 51 (13.1) C low literacy: 59 (10.4) C high literacy: 56 (10.9)	I low literacy: 45 I high literacy: 35 C low literacy: 47 C high literacy: 42	One-to-one educational sessions including counseling and medication management Communication individualized depending on patients literacy status	Intensive diabetes management from three clinical pharmacist practitioners and a diabetes care coordinator (DCC)	Patients contacted every two to four weeks by telephone or in person by pharmacist or DCC	Application of evidence-based treatment algorithms to help manage glucose and cardiovascular risk
Sperl-Hillen and O’Connor (2005)	USA	RC	112	5610–7650	59–61	52–54	Nurses provided diabetes education and self-management training	Diabetes education nurses work closely with primary care physicians	Patient registry. Nurses use the registries to guide ‘active outreach’ to high-risk patients not in metabolic control or missing recommended tests	Drug formulary facilitated use of sulfonylureas, metformin, insulin, fibrates and 3-hydroxy-3-methyl-glutaryl-coenzyme A reductase inhibitors
Taweepolcharoen *et al*. (2006)	TH	CS	N/A	1510	58.8 (10.9)	34.6	Group diabetes education supervised by registered nurses and dieticians	Clinic is served by three groups of working physicians, consisting of faculty members, family medicine residents and service GPs. There are also registered nurses and dieticians		
Trief *et al*. (2006)	USA	CT	12	1665	70.8 (6.6)	37.2	Nurse case manager provided diabetes education	Nurse case manager provides, under the supervision of an endocrinologist, treatment planning and consultation to PCPs who maintained decision authority for their patients A separate team of trained research nurses conducts physical and psychological assessments at baseline and one-year follow-up	Intervention subjects received a home telemedicine unit, ie, a web-enabled computer used to upload blood pressure and blood glucose measurements, to videoconference with a nurse case manager and dietician, and to access individualized graphic data displays and educational materials	
Uitewaal *et al*. (2004)	NL	RC	24	T: 106 D: 90	T: 50.5 (7.5) D: 55.3 (8.2)	T: 43.3 D: 51.1		Four visits to the GP per year Blood glucose and weight are measured at every visit. Other blood measures and feet and eye inspection every year	Computer-based patient records	Guideline recommending four visits to the GP per year
Uitewaal *et al*. (2005)	NL	CT	12	I: 53 C: 51	I: 50.6 (9.3) C: 53.5 (6.2)	I: 40 C: 38	Culturally acceptable and ethnic specific diabetes program for Turkish diabetes patients, consisting of seven individual education sessions and three group sessions Program was based on three principles: peer education, tailoring and the Health Education Model	Individual sessions consisting of four sessions with the educator and patient together and three ‘triangle’ sessions with the GP, educator and patient present, to discuss three-monthly assessment of glycemic control and cardiovascular risk factors Patients were encouraged to have one of the individual sessions with the dietician and one with the partner present, although this was not obligatory	Computer-based patient records	
Whaba and Chang (2007)	USA	CS	N/A	136	59.7 (15.2)	51.5	Individual care plan Self-monitoring of blood glucose	Care team (dietitian, DM nurse educator and physician) Patient referred to ophthalmologic and podiatric evaluations as soon as the diagnosis of DM was made Regular follow-up	Patient prescribed a glucose meter and advised to keep a diary of those readings to share with the physician at each office visit	Plan of care developed specifically for the patient’s clinical condition Laboratory tests were conducted at least twice a year Compliance with diet and medications was assessed at each visit A DM flow sheet was created for each patient to keep track of the laboratory values, medications, and immunizations

CCM=chronic care model; Jor=Jordan; CS=cross-sectional; N/A=not applicable; RC=retrospective cohort; ES=Spain; PC=prospective cohort; DK=Denmark; NL=the Netherlands; Mex=Mexico; RCT=randomized controlled trials; AUS=Australia; CH=China; SWE=Sweden; HbA1c=hemoglobin A1c; SG=Singapore; TH=Thailand; CT=controlled trial; PCP=prospective cohort physician; T=Turkish; D=Dutch; DM=diabetes mellitus

Table 2 also provides an overview of the CCM components implemented in each study. Eight studies included all four components of the CCM model. The CCM component delivery system design was included in most studies (*n*=25), followed by self-management support (*n*=20). Of the studies that included the components delivery system design, most introduced a care team (*n*=13), followed by regular follow-up visits (*n*=8). Self-management support was mostly realized through individual educational sessions on diabetes, health and nutrition (*n*=14).

### Outcome variables

#### HbA1c

In total, 18 uncontrolled studies – including prospective, retrospective and cross-sectional cohort designs – measured the effects of integrated care programs on HbA1c. In addition, seven studies compared the influence of patient characteristics on the effectiveness of integrated diabetes care programs between intervention and control groups. In total, 51 patient characteristics were assessed as potential effect modifiers of the relationship between integrated care and HbA1c. The results will be presented according to study design. For RCTs all characteristics assessed by this study design will be discussed. Due to the high number of characteristics assessed by the cross-sectional, retrospective and prospective cohort studies, only characteristics assessed by three or more studies will be presented.

(Randomized) controlled trials: Five RCTs and two controlled trials (CTs) compared the influence of patient characteristics on the effectiveness of integrated diabetes care programs on the HbA1c level between intervention and control groups (Table 3). In total, eight patient characteristics were evaluated as potential modifiers.

**Table 3:** Subgroup intervention effects on hemoglobin A1c (HbA1c)

	Variables entered in multivariate regression model	Global quality rating	Person-related characteristics					
Study			Female	Male	Lower age^a^	Higher age^b^		
Nielsen *et al*. (2006)	Clustering effect at the general practitioner level, interaction between age and baseline HbA1c, DM duration, BMI, number of DM-related consultations, interaction between the patients’ physical activity level, antidiabetic medication and dietary habits	Weak	−	o				
Uitewaal *et al*. (2005)^c^	Baseline HbA1c, sex, age, DM duration, DM medication, indicators of DM care	Weak	−	o				
Moreira *et al*. (2015)	N/A	Weak	o	o	−	o		
Quinn *et al*. (2016)	Study group, time, age, all two-way interactions and three-way interaction	Moderate			−	−		
			Context-related characteristics					
			Low literacy status	High literacy status	Monthly income ⩽$118 26	Monthly income >$118 26	⩽Four years of schooling	>Four years of schooling
Rothman *et al*. (2004)	Baseline HbA1c, age, race, sex, income, DM medication, DM duration, income	Weak	−	o				
Moreira *et al*. (2015)	N/A	Weak			−	o	−	o
			Health-related characteristics					
			FBG >10 mmol/L	FBG ⩽10 mmol/L	Depression Yes	Depression No	DM duration <five years	DM duration ⩾five years
Groeneveld *et al*. (2001)	N/A	Weak	−	o				
Trief *et al*. (2006)	Baseline HbA1c, ethnicity, age, sex, marital status, years of education, DM duration, insulin use, smoking, co-morbidity, clustering effect at the general practitioner level	Weak			o	o		
Moreira *et al*. (2015)	N/A	Weak					−	o

DM=diabetes mellitus; BMI=body mass index; N/A: not applicable; FBG=fasting blood glucose.

a Lower age: ⩽52 years (Moreira *et al*., 2015), <55 years (Quinn *et al*., 2016).

b Higher age: >52 years (Moreira *et al*., 2015), ⩾55 years (Quinn *et al*., 2016).

c Intervention and control groups only consisted of patients with a baseline HbA1c >7%.

o: No significant relationship between the characteristic with HbA1c for people in the intervention group compared to usual care; −: significant negative relationship between the characteristic with HbA1c for patients in the intervention group compared to usual care.

Sex and age were the person-related characteristics evaluated as potential effect modifiers. Three studies assessed sex as a potential modifier, of which two found that women in the intervention group had statistically significant lower HbA1c values at follow-up compared to women in the control group (Uitewaal *et al*., 2005; Nielsen *et al*., 2006). For men, no statistically significant difference was found. The third study did not find a statistically significant relationship (Moreira *et al*., 2015). Age was assessed by two studies. Both found that younger patients receiving integrated diabetes care had statistically significantly lower HbA1c values at follow-up compared to patients receiving usual care (Moreira *et al*., 2015; Quinn *et al*., 2016).

Three health-related characteristics were evaluated as potential effect modifiers of the relationship between integrated diabetes care programs and HbA1c: literacy status, income and number schooling years. Literacy status was assessed by one study (Rothman *et al*., 2004), which found that patients in the intervention group with low literacy status (⩽6th grade) had statistically significant lower HbA1c values at follow-up compared to patients with low literacy status receiving usual care. Monthly income and number of schooling years were also each assessed by one study. Patients with lower monthly income (⩽$118.26) and ⩽four years of schooling at baseline receiving integrated diabetes care had significantly lower HbA1c values at follow-up compared to patient receiving usual care (Moreira *et al*., 2015).

Three health-related characteristics were evaluated as potential effect modifiers of the relationship between integrated diabetes care programs and HbA1c: fasting blood glucose (FBG), depression and diabetes mellitus (DM) duration. Each characteristic was assessed by one study. Patients with high FBG (>10 mmol/L) at baseline receiving integrated diabetes care had significantly lower HbA1c levels at follow-up compared to patients receiving usual care (Groeneveld *et al*., 2001). For patients with a FBG ⩽10 mmol/L no significant difference was found in HbA1c levels at follow-up between the intervention and control groups. Depression was not an effect modifier of the association between integrated diabetes care programs and HbA1c (Trief *et al*., 2006). Patients with a DM duration <five years receiving integrated diabetes care had significantly lower HbA1c levels at follow-up compared to patients receiving usual care (Moreira *et al*., 2015).

No RCTs assessed context-related characteristics as potential effect modifiers of the relationship between integrated diabetes care programs and HbA1c.

Prospective and retrospective cohort studies: In total, 11 prospective and retrospective cohort studies measured the effects of integrated diabetes care programs on HbA1c (Tables 4 and 5). Three studies compared the change in HbA1c between levels of patient characteristics (Rothman *et al*., 2003; Sperl-Hillen and O’Connor, 2005; Elissen *et al*., 2012). The other eight studies compared HbA1c levels at follow-up between levels of patient characteristics (El-Kebbi *et al*., 2003; Benoit *et al*., 2005; Mold *et al*., 2008; De Fine Olivarius *et al*., 2009; Robinson *et al*., 2009; Kellow *et al*., 2011; Cardenas-Valladolid *et al*., 2012; LeBlanc *et al*., 2015).

**Table 4:** Relationship between hemoglobin A1c (HbA1c) and person-related and context-related characteristics

			Person-related characteristics							
			Socio-demographics					Lifestyle		Context-related characteristic
Study	Variables entered in multivariate regression model	Global quality rating	Age	Sex^a^	Ethnicity	Marital status^b^	Education	BMI	Smoking	Health insurance
Prospective cohort studies										
Cardenas-Valladolid *et al*. (2012)	Age, sex, DM medication	Moderate	o	+						
De Fine Olivarius *et al*. (2009)	Age, sex, BMI, HbA1c baseline, SBP, TC, urinary albumin	Moderate	o	o				o		
Retrospective cohort studies										
Benoit *et al*. (2005)	A1c, time, age, TC, DM duration, Medication	Strong	−	o	o^c^			o	o^d^	o^e^
Sperl-Hillen and O’Connor (2005)	Age, sex, baseline HbA1c, DM medication, depression, co-morbidities, PC physician variable (age, sex, specialty), diabetes educator visits, pharmacy coverage	Weak	+	o						−^f^
Elissen *et al*. (2012)	N/A	Weak	−						+^g^	
El-Kebbi *et al*. (2003)	Year of presentation, age, sex, ethnicity, BMI, DM duration, baseline HbA1c, DM medication, no. of interval visits, follow-up duration	Strong	−	o	o^h^			+		
LeBlanc *et al*. (2015)	Age, sex, DM duration, DM medication, Charlson co-morbidity index	Strong	−	o						
Kellow, Savige and Khalil (2011)	Age, sex, OGTT, HbA1c, TC, HDL, TG, LDL/HDL ratio, weight change, body weight	Moderate	−	o				o	o^i^	
Mold *et al*. (2008)	N/A	Moderate	−		+^j^					
Robinson *et al*. (2009)	N/A	Weak	o	o	o^k^	o				o^l^
Rothman *et al*. (2003)	Age, sex, ethnicity, education, insurance, BMI, HbA1c, DM medication, hypertension medication, hypercholesterolemia medication, recent diagnosis of DM, DM duration	Moderate	o	o	o^m^		o^n^	o		O^o^
Cross-sectional studies										
Al Omari *et al*. (2009)	DM medication, DM duration	Weak	o	o				o	o^p^	
De Alba Garcia *et al*. (2006)	Age, sex, marital status, education, BMI, smoking, follow diet, glucose, family history of DM, DM duration, DM medication, SBP, DBP, TC, TG	Weak	o	o		o	o^q^	o	o^r^	
Ostgren *et al*. (2002)	Age, sex, waist–hip ratio, TG, *β*-cell function	Weak	o					o		
Quah *et al*. (2013)	Age, sex, ethnicity, marital status, occupation, housing type, DM duration, DM medication, compliance to medication, self-monitoring, BMI	Moderate	−	o	o^s^	o	o^t^	o	o^u^	
Taweepolcharoen *et al*. (2006)	Age, sex, DM duration, BMI, BP, fasting glucose, TG, HDL, LDL	Weak	o	+				o		
Whaba and Chang (2007)	Age, DM duration, BMI, DM medication, hypertension, hyperlipidemia	Moderate	−	o				o		

BMI=body mass index; DM=diabetes mellitus; SBP=systolic blood pressure; TC=total cholesterol; PC=prospective cohort; N/A=not applicable; OGTT=oral glucose tolerance test; HDL=high-density lipoprotein; LDL=low-density lipoprotein; TG=triglycerides; BP=blood pressure.

a 0=male, 1=female.

b 0=not married, 1=married.

c 0=Hispanic, black and white, 1=Asian.

d 0=current smoker, 1=past smoker, 2=never smoker.

e 0=insured, 1=County Medical Services, 3= uninsured.

f 0=pharmacy coverage, 1=no pharmacy coverage.

g 0=current smoker, 1=none smoker/previous smoker.

h 0=others, 1=African American.

i 0=non-smoker, 1= current smoker.

j 0=white, 1=black Caribbean/African.

k 0=white, 1=Asian, 2=black, 3=other.

l 0=insured, 1=uninsured.

m 0=black, 1=others.

n 0=less than high school, 1=high school or higher.

o 0=Medicaid or pharmacy assistance programs, 1= no Medicaid or pharmacy assistance program.

p 0=current smoker, 1=past and none smoker.

q 0=none, 1=incomplete primary, 2=completed primary, 3=primary.

r 0=smoker, 1=none smoker.

s 0=Chinese, 1=Malay, 2=Indian, 3=others.

t 0=no formal education, 1=formal education.

u 0=none smoker, 1=past smoker, 2=current smoker.

+: positive significant relationship; o- non-significant relationship; −: significant negative relationship.

**Table 5:** Relationship between hemoglobin A1c (HbA1c) and health-related characteristics

			Health-related characteristics										
Study	Variables entered in multivariate regression model	Global quality rating	HbA1c	SBP	DBP	TC	HDL	LDL	TG	# Providers visits	DM duration	Medication^a^	# Co-morbidities
Prospective cohort studies													
Cardenas-Valladolid *et al*. (2012)	Age, sex, DM medication	Moderate											
De Fine Olivarius *et al*. (2009)	Age, sex, BMI, HbA1c baseline, SBP, TC, urinary albumin	Moderate		o		o						+	
Retrospective cohort studies													
Benoit *et al*. (2005)	A1c, time, age, TC, DM duration, Medication	Strong	+	o	o	+	o			o	+	+	
Sperl-Hillen and O’Connor (2005)	Age, sex, baseline HbA1c, DM medication, depression, co-morbidities, PC physician variable (age, sex, specialty), diabetes educator visits, pharmacy coverage	Weak	+									+	o
Elissen *et al*. (2012)	N/A	Weak	+								+		+
El-Kebbi *et al*. (2003)	Year of presentation, age, sex, ethnicity, BMI, DM duration, baseline HbA1c, DM medication, no. of interval visits, follow-up duration	Strong	+							−	+	+	
Kellow, Savige and Khalil (2011)	Age, sex, OGTT, HbA1c, TC, HDL, TG, LDL/HDL ratio, weight change, body weight	Moderate	o	o	o	o	o	o	o			o	o
LeBlanc *et al*. (2015)	Age, sex, DM duration, DM medication, Charlson co-morbidity index	Strong	+								+	+	
Mold *et al*. (2008)	N/A	Moderate								−	+	+	
Robinson *et al*. (2009)	N/A	Weak											
Rothman *et al*. (2003)	Age, sex, ethnicity, education, insurance, BMI, HbA1c, DM medication, hypertension medication, hypercholesterolemia medication, recent diagnosis of DM, DM duration	Moderate	+								−	o	
Cross-sectional studies													
Al Omari *et al*. (2009)	DM medication, DM duration	Weak				o	o	o	o		+	+	
De Alba Garcia *et al*. (2006)	Age, sex, marital status, education, BMI, smoking, follow diet, glucose, family history of DM, DM duration, DM medication, SBP, DBP, TC, TG	Weak		o	o	o			o	o	+	+	
Ostgren *et al*. (2002)	Age, sex, waist–hip ratio, TG, *β*-cell function	Weak		−	−	o			+		o		
Quah *et al*. (2013)	Age, sex, ethnicity, marital status, occupation, housing type, DM duration, DM medication, compliance to medication, self-monitoring, BMI	Moderate									+	+	o
Taweepolcharoen *et al*. (2006)	Age, sex, DM duration, BMI, BP, fasting glucose, TG, HDL, LDL	Weak					o	o	o		+		
Whaba and Chang (2007)	Age, DM duration, BMI, DM medication, hypertension, hyperlipidemia	Moderate									o	o	o

SBP=systolic blood pressure; DBP=diastolic blood pressure; TC=total cholesterol; HDL=high-density lipoprotein; LDL=low-density lipoprotein; TG=triglycerides; DM=diabetes mellitus; PC=primary care; OGTT=oral glucose tolerance test; N/A=not applicable; BMI=body mass index; BP=blood pressure. +: positive significant relationship; o: non-significant relationship; −: significant negative relationship.

a 0=no insulin, 1=insulin.

Most examined person-related characteristics were age (*n*=11) and sex (*n*=9). In seven studies the effect of integrated diabetes care programs on HbA1c differed significantly across ranges of age: younger patients had higher HbA1c levels at follow-up compared to older patients (*n*=5) and experienced greater change from baseline in HbA1c (*n*=2) (El-Kebbi *et al*., 2003; Benoit *et al*., 2005; Sperl-Hillen and O’Connor, 2005; Mold *et al*., 2008; Kellow *et al*., 2011; Elissen *et al*., 2012; LeBlanc *et al*., 2015). As to the latter, the direction of the measured change in HbA1c differed: one study found a significant improvement (Sperl-Hillen and O’Connor, 2005) and the other a significant increase (Elissen *et al*., 2012) in HbA1c. Age was not a significant effect modifier in the other four studies (Rothman *et al*., 2003; De Fine Olivarius *et al*., 2009; Robinson *et al*., 2009; Cardenas-Valladolid *et al*., 2012). The effect of integrated care on HbA1c did not differ between men and women in eight studies (El-Kebbi *et al*., 2003; Rothman *et al*., 2003; Benoit *et al*., 2005; Sperl-Hillen and O’Connor, 2005; De Fine Olivarius *et al*., 2009; Robinson *et al*., 2009; Kellow *et al*., 2011; LeBlanc *et al*., 2015). In one study females had significantly higher HbA1c levels at follow-up compared to males (Cardenas-Valladolid *et al*., 2012).

Most examined health-related characteristics were medication use (*n*=8), baseline HbA1c (*n*=7) and duration of type 2 diabetes (*n*=6). The effect of integrated diabetes care programs on HbA1c was different for people on insulin therapy. These patients had higher HbA1c levels at follow-up compared with patients on diet and/or oral therapy in five studies(El-Kebbi *et al*., 2003; Benoit *et al*., 2005; Mold *et al*., 2008; De Fine Olivarius *et al*., 2009; LeBlanc *et al*., 2015) and less desirable changes in HbA1c from baseline (Sperl-Hillen and O’Connor, 2005). In two studies the relationship between integrated diabetes care programs and HbA1c did not differ between types of medication (Rothman *et al*., 2003; Kellow *et al*., 2011). In the studies assessing baseline HbA1c, patients with higher baseline HbA1c levels had higher HbA1c levels at follow-up (*n*=3) (El-Kebbi *et al*., 2003; Benoit *et al*., 2005; LeBlanc *et al*., 2015), but did have greater improvements in HbA1c from baseline (*n*=3) (Rothman *et al*., 2003; Sperl-Hillen and O’Connor, 2005; Elissen *et al*., 2012) compared to patients with lower baseline HbA1C levels. In one study baseline HbA1c was not a significant effect modifier (Kellow *et al*., 2011). The effect of integrated diabetes care programs on HbA1c differed significantly across ranges of diabetes duration in five studies. Patients with longer diabetes duration had significantly higher HbA1c levels at follow-up compared to patients with shorter diabetes duration (*n*=5) (El-Kebbi *et al*., 2003; Benoit *et al*., 2005; Mold *et al*., 2008; Elissen *et al*., 2012; LeBlanc *et al*., 2015). In one study a significant opposite effect was found (Rothman *et al*., 2003).

Health insurance status was assessed by four studies. It did not seem to significantly modify the observed effect of integrated care on HbA1c in three studies (Rothman *et al*., 2003; Benoit *et al*., 2005; Robinson *et al*., 2009). Patients with no health insurance coverage had less desirable changes in HbA1c than those with health insurance coverage (Sperl-Hillen and O’Connor, 2005). No other context-related characteristics were examined by the included studies.

Cross-sectional studies: In total, six cross-sectional studies measured the modifying effect of patient characteristics on the relationship between integrated diabetes care programs and HbA1c (Tables 4 and 5).

Most examined person-related characteristics were age (*n*=6), body mass index (BMI) (*n*=6) and sex (*n*=5). Four studies of integrated care programs found non-significant associations between age and HbA1c (Ostgren *et al*., 2002; De Alba Garcia *et al*., 2006; Taweepolcharoen *et al*., 2006; Al Omari *et al*., 2009). In two studies significant associations were found: in these studies, younger patients had higher HbA1c levels (Wahba and Chang, 2007; Quah *et al*., 2013). The effect of integrated diabetes care programs on HbA1c did not significantly differ between levels of BMI in all studies (Ostgren *et al*., 2002; De Alba Garcia *et al*., 2006; Taweepolcharoen *et al*., 2006; Wahba and Chang, 2007; Al Omari *et al*., 2009; Quah *et al*., 2013). The effect on HbA1c did also not differ between men and women in four studies (De Alba Garcia *et al*., 2006; Wahba and Chang, 2007; Al Omari *et al*., 2009; Quah *et al*., 2013). In one study females had significantly higher HbA1c levels compared to males (Taweepolcharoen *et al*., 2006).

Most examined health-related characteristics were duration of type 2 diabetes (*n*=6) and medication use (*n*=4). The effect of integrated care programs on HbA1c differed significantly across ranges of diabetes duration in four studies (De Alba Garcia *et al*., 2006; Taweepolcharoen *et al*., 2006; Al Omari *et al*., 2009; Quah *et al*., 2013). Patients with longer diabetes duration had higher HbA1c levels compared to patients with shorter diabetes duration in these studies. In two studies diabetes duration was not a significant effect modifier (Ostgren *et al*., 2002; Wahba and Chang, 2007). The effect of integrated care programs on HbA1c was also different for people on insulin therapy. These patients had higher HbA1c concentrations compared with patients on diet and/or oral therapy in three studies (De Alba Garcia *et al*., 2006; Al Omari *et al*., 2009; Quah *et al*., 2013). In one study type of medication was not a significant effect modifier (Wahba and Chang, 2007).

No context-related characteristics were assessed by three or more studies.

#### LDL-c

Three prospective and retrospective cohort studies measured the effect of integrated diabetes care programs on LDL-c. The RCTs and cross-sectional studies included in this review did not measure this effect. In total, 11 patient characteristics were assessed by the studies. Only those results that were assessed by at least two studies will be discussed.

Prospective and retrospective cohort studies: The person-related characteristic age was examined by three studies (Sperl-Hillen and O’Connor, 2005; Robinson *et al*., 2009; Elissen *et al*., 2012). The relationship between age and LDL-c was inconsistent: a negative and positive as well as a non-significant relationship were found.

The modifying effect of baseline LDL-c on the relationship between integrated diabetes care programs and changes in LDL-c over baseline was assessed by two studies (Sperl-Hillen and O’Connor, 2005; Elissen *et al*., 2012). Both found that patients with higher baseline LDL-c had greater LDL-c improvements.

No context-related characteristics were assessed by the included studies.

#### SBP

Four retrospective and prospective cohort studies measured the effect of integrated diabetes care programs on SBP. In total, nine patient characteristics were assessed by the studies. Only those results that were assessed by at least two studies will be discussed.

Retrospective cohort and prospective cohort studies: Age was measured by three studies (Mold *et al*., 2008; Robinson *et al*., 2009; Elissen *et al*., 2012). These studies found that higher age was associated with higher SBP at follow-up (Mold *et al*., 2008; Robinson *et al*., 2009) and greater improvement (Elissen *et al*., 2012). The modifying effect of ethnicity on integrated care programs and SBP was measured by two studies (Mold *et al*., 2008; Robinson *et al*., 2009). The effect was unclear, as results were inconsistent between these studies. Four other characteristics were assessed, one context-related and three health-related characteristics, by one study each.

#### Health-care utilization

Health-care utilization was assessed by three studies: one RCT (Nielsen *et al*., 2006), one retrospective cohort study (Uitewaal *et al*., 2004) and one cross-sectional study (Liu *et al*., 2013). Together they measured the modifying effect of integrated care programs and health-care utilization for five person-related characteristics, one context-related characteristic and one health-related characteristic. Most examined characteristic was sex, which was measured by two studies (Nielsen *et al*., 2006; Liu *et al*., 2013). Nielsen *et al*. (2006) found that females in the intervention group had statistically significant more GP consultations per year compared to females in the control group (Nielsen *et al*., 2006). For males, no difference was found. Liu *et al*. found that the effect of integrated diabetes care programs on health-care utilization was different between males and females (Liu *et al*., 2013). Females had higher utilization of community health centers compared to male.

## Discussion

This paper presents a literature review on relevant patient characteristics for guiding tailored integrated type 2 diabetes care in primary care. HbA1c was considered an outcome in 93% of the 27 studies identified. Many different patient characteristics were investigated by these studies. Findings indicate that the effect of integrated primary care programs on HbA1c differs significantly according to a number of person and health-related characteristics. Younger age, longer disease duration, higher baseline HbA1c and insulin therapy were associated with higher HbA1c levels. Health insurance status, living situation and income were the only context-related characteristics in the included studies and were not frequently assessed.

Compared to HbA1c, LDL-c, SBP and health-care utilization were included far less. It was found that higher baseline LDL-c lead to greater LDL-c improvement. Patients with higher age had higher SBP levels at follow-up as well as greater improvements in SBP compared to younger patients. The relationship between integrated care and health-care utilization seemed to be modified by sex: women had more consultations per year compared to men.

Several factors might explain the elevated HbA1c levels in a subset of patients with type 2 diabetes. Younger patients tend be more non-adherent to oral medication therapy and experience less profound diabetes-related health problems than older patients (Pyatak *et al*., 2014; Tunceli *et al*., 2015). The latter might cause them to believe that a proactive attitude toward their disease is less important. Moreover, younger patients and/or those with longer disease duration undergo a more rapid decline in *β* cell function and pancreatic insulin secretion, resulting in the need for a more complex and intensive drug therapy (Al Omari *et al*., 2009; Fonseca, 2009; Khattab *et al*., 2010; Kellow *et al*., 2011). Higher HbA1c levels for patients on insulin therapy compared to patients on diet and/or oral therapy could be due to a delayed start or low intensity of insulin therapy (Abraira *et al*., 1995; El-Kebbi *et al*., 2003; Mosenzon and Raz, 2013). Furthermore, maintaining glycemic control, while minimizing hypoglycemia and sticking to a diet might be difficult (Jin *et al*., 2008; Quah *et al*., 2013).

High HbA1c at baseline also seemed to be predictive of later HbA1c. First, type 2 diabetes is a heterogeneous disease in both pathogenesis and clinical manifestation (Inzucchi *et al*., 2012), thus a high HbA1c at baseline and at follow-up could be due to decreased insulin sensitivity, secretion and *β*-cell dysfunction (Heianza *et al*., 2012). Second, unhealthy lifestyle habits, such as low physical activity, and a diet rich in carbohydrates have been associated with less glycemic control (Mozaffarian *et al*., 2009; Inzucchi *et al*., 2012). Changing these lifestyle factors is easier said than done, making it difficult for patients to improve their glycemic control.

Several factors could explain the differences in levels of LDL-c, SBP and health-care utilization between levels of patient characteristics. Prescription of statins usually follows when LDL-c level is 2.5 mmol/L or higher, possibly leading to greater improvements in LDL-c for those patients with high baseline LDL-c levels (The Dutch college of general practitioners, 2011). The higher SBP levels at follow-up for older patients may be due to less stringent treatment targets (van Hateren *et al*., 2012; James *et al*., 2014). The greater health-care utilization by women compared to men might be explained by the difference in perception of illness between men and women. According to some studies, it is more culturally and socially accepted for women to be ill than it is for men (De Visser *et al*., 2009).

Overall, our results indicate the need to implement integrated diabetes care programs specifically tailored to the needs, values and preferences of younger patients and to those on insulin therapy, with longer disease duration and/or higher HbA1c levels and older patients with high SBP levels. These effect modifiers can help to provide the right care to the right person at the right time. At this moment, not every patient with these characteristics receives such care. Current practice might therefore not be suitable for all patients. Lack of motivation, family support and feeling burned-out from managing diabetes are reported barriers to optimal self-management (Browne *et al*., 2013). To tackle these barriers, diabetes treatment programs should take them into account by, for example, providing shared decision making and simple and specific instructions and advice, involving family members and offering online consultations or evening primary care opening hours. In addition to patients who find it difficult to keep their diabetes under control, there is a large group of patients who does manage to control their diabetes (Rothe *et al*., 2008; Elissen *et al*., 2012). For these patients, fewer visits to primary care might have similar outcomes and thus should be taken into consideration by both the GP and the patient. Allowing care givers to provide care based on patient characteristics constitutes a promising approach for achieving the so-called ‘Triple Aim’ by: (1) improving patient experience, by including patients’ care needs, preferences, and abilities in treatment decisions; (2) improving population health and quality of life, by supporting tailored diabetes care; and (3) reducing the per capita cost of diabetes care, by reducing the over-, under- and misuse of health-care services (Berwick *et al*., 2008).

This review has several limitations that should be taken into account. First, given the scarceness of studies assessing the differences in the effect of integrated diabetes care programs on diabetes control measures by levels of patient characteristics, it was decided to include RCTs, prospective and retrospective cohort studies. However, this introduced significant heterogeneity and made it impossible to conduct a meta-analysis. Second, quality of the studies was weak for most studies. This was mainly due to the cross-sectional study design of more than one-third of the studies and the use of less robust statistical methods. Fortunately, it is unlikely that these studies altered the results, as their findings were similar to those of the other, more robust studies. Third, very few context- and person-related characteristics were analyzed. Studies performed in a non-integrated diabetes care setting, found that context-related characteristics, such as socio-economic status and social network, are associated with measures of diabetes control and are likely to be strong predictors of diabetes control (Jotkowitz *et al*., 2006; Nam *et al*., 2011). Person-related characteristics, such as low mastery and low self-efficacy, have been related to negative health outcomes (Bosma *et al*., 2014; Elissen *et al*., 2017). Traditionally, researchers and care providers have looked at diabetes from a mostly biomedical viewpoint, which might explain the relatively scarce collection of context- and person-related characteristics in routinely collected individual patient data (Hasnain-Wynia and Baker, 2006).

The current review provides a good understanding of which characteristics can help to identify patients with different health-care needs and preferences. However, to implement an effective integrated type 2 diabetes tailored care program, it is necessary to know which context- and person-related characteristics are important to identify patients. Furthermore, implementation of an effective tailored diabetes care program is only possible by taking into account the care preferences of patients and caregivers. In the next phase of the PROFILe project (Elissen *et al*., 2016), data rich in non-health-related characteristics will be analyzed to assess which of these are predictors of diabetes control measures and a discrete choice experiment will be conducted to gain knowledge on patients’ care preferences as a first step toward patient-centered diabetes care.

## Supplementary material

For supplementary material accompanying this paper visit https://doi.org/10.1017/S146342361800004X. click here to view supplementary material
