# Supplementary material for: Relevant patient characteristics for guiding tailored integrated diabetes primary care: a systematic review
Source: Prim Health Care Res Dev. 2018 Feb 6;19(5):424–47. doi: 10.1017/S146342361800004X (PMC6452927; doi:10.1017/S146342361800004X)
Supplement: Supplementary file 1 [file S146342361800004Xsup001.docx]

**Supplementary material**

**Supplementary Table S1.** Quality of included studies

| **Study** | **Study design** | **Selection bias** | **Study design** | **Confounders** | **Blinding** | **Data collection** | **Drop-outs** | **Global** |
| --- | --- | --- | --- | --- | --- | --- | --- | --- |
| Al Omari et al. (2009) | CS | + | - | - | o | + | NA | - |
| Benoit et al. (2005) | RC | + | o | + | o | + | NA | + |
| Cardenas-Valladolid et al. (2012) | PC | + | o | + | o | + | - | o |
| De Fine Olivarius et al. (2009) | PC | + | o | + | - | + | + | o |
| Elissen et al. (2012) | RC | o | o | - | + | + | - | - |
| El Kebbi et al. (2003) | RC | o | o | + | + | + | NA | + |
| De Alba Garcia et al. (2006) | CS | - | - | + | - | + | + | - |
| Groeneveld et al. (2001) | RCT | o | + | - | - | + | o | - |
| Kellow, SavigeandKhalil(2011) | RC | o | o | o | + | + | - | o |
| LeBlanc et al (2015) | RC | o | o | + | + | + | NA | + |
| Liu et al. (2013) | CS | + | - | o | - | o | NA | - |
| Luijks et al. (2015) | PC | + | o | + | o | + | + | + |
| Mold, WhileandForbes (2008) | RC | o | o | - | + | + | NA | o |
| Moreira et al. (2015) | RCT | - | + | - | o | + | + | - |
| Nielsenet al. (2006) | RCT | - | + | + | - | + | - | - |
| Ostgren et al. (2002) | CS | + | - | - | - | + | NA | - |
| Quah et al. (2013) | CS | o | - | + | o | o | NA | o |
| Quinn et al. (2016) | RCT | o | + | - | o | + | o | o |
| Robinson et al. (2009) | PC | - | o | - | - | + | - | - |
| Rothman et al. (2003) | RC | - | o | + | o | + | + | o |
| Rothman et al. (2004) | RCT | o | + | + | - | + | + | o |
| Sperl Hillen and O’Connor (2005) | RC | + | - | o | o | + | + | o |
| Taweepolcharoen et al. (2006) | CS | + | - | - | + | + | NA | - |
| Trief et al. (2006) | CT | - | o | + | + | + | + | o |
| Uitewaal et al. (2004) | RC | - | o | - | o | + | NA | - |
| Uitewaal et al. (2005) | CT | - | + | + | - | + | + | - |
| WhabaandChang (2007) | CS | - | o | o | o | + | NA | o |
| Sum  (+/o/-) |  | 9/9/9 | 6/14/7 | 13/4/10 | 7/11/9 | 25/2/0 | 9/2/5 | 4/11/12 |

Abbreviations: CS: cross-sectional; RC: retrospective cohort; PC: prospective cohort; RCT: randomized controlled trial; CT: controlled trial

+: strong; o: moderate; -:weak
